# Regular use of analgesics is a risk factor for renal cell carcinoma

**DOI:** 10.1038/sj.bjc.6690728

**Published:** 1999-10

**Authors:** M Gago-Dominguez, J-M Yuan, J E Castelao, R K Ross, M C Yu

**Affiliations:** Department of Preventive Medicine, USC/Norris Comprehensive Cancer Center, M/S #44, University of Southern California, Los Angeles, CA 90033-0800, USA

**Keywords:** renal cell cancer, analgesics, NSAID, aspirin, acetaminophen, phenacetin

## Abstract

Phenacetin-based analgesics have been linked to the development of renal pelvis cancer and renal cell carcinoma (RCC). The relationship between non-phenacetin types of analgesics and kidney cancer is less clear, although laboratory evidence suggests that these drugs possess carcinogenic potential. A population-based case–control study involving 1204 non-Asian RCC patients aged 25–74 and an equal number of sex-, age- and race-matched neighbourhood controls was conducted in Los Angeles, California, to investigate the relationship between sustained use of analgesics and risk of RCC according to major formulation categories. Detailed information on medical and medication histories, and other lifestyle factors was collected through in-person interviews. Regular use of analgesics was a significant risk factor for RCC in both men and women (odds ratio (OR) = 1.6, 95% confidence interval (CI) = 1.4–1.9 for both sexes combined). Risks were elevated across all four major classes of analgesics (aspirin, non-steroidal anti-inflammatory agents other than aspirin, acetaminophen and phenacetin). Within each class of analgesics, there was statistically significant increasing risk with increasing level of exposure. Although there was some minor variability by major class of formulation, in general individuals in the highest exposure categories exhibited approximately 2.5-fold increase in risk relative to non- or irregular users of analgesics. Subjects who took one regular-strength (i.e. 325 mg) aspirin a day or less for cardiovascular disease prevention were not at an increased risk of RCC (OR = 0.9, 95% CI = 0.6–1.4). © 1999 Cancer Research Campaign


*British Journal of Cancer* (1999) **81**(3), 542–548
© 1999 Cancer Research Campaign
Article no. bjoc.1999.0728

### Regular use of analgesics is a risk factor for renal cell carcinoma

**M Gago-Dominguez, J-M Yuan, JE Castelao, RK Ross and MC Yu**

Department of Preventive Medicine, USC/Norris Comprehensive Cancer Center, M/S #44, University of Southern California, 1441 Eartlake Avenue, Los Angeles, CA 90033-0800, USA

**Summary** Phenacetin-based analgesics have been linked to the development of renal pelvis cancer and renal cell carcinoma (RCC). The relationship between non-phenacetin types of analgesics and kidney cancer is less clear, although laboratory evidence suggests that these drugs possess carcinogenic potential. A population-based case–control study involving 1204 non-Asian RCC patients aged 25–74 and an equal number of sex-, age- and race-matched neighbourhood controls was conducted in Los Angeles, California, to investigate the relationship between sustained use of analgesics and risk of RCC according to major formulation categories. Detailed information on medical and medication histories, and other lifestyle factors was collected through in-person interviews. Regular use of analgesics was a significant risk factor for RCC in both men and women (odds ratio (OR) = 1.6, 95% confidence interval (CI) = 1.4–1.9 for both sexes combined). Risks were elevated across all four major classes of analgesics (aspirin, non-steroidal anti-inflammatory agents other than aspirin, acetaminophen and phenacetin). Within each class of analgesics, there was statistically significant increasing risk with increasing level of exposure. Although there was some minor variability by major class of formulation, in general individuals in the highest exposure categories exhibited approximately 2.5-fold increase in risk relative to non- or irregular users of analgesics. Subjects who took one regular-strength (i.e. 325 mg) aspirin a day or less for cardiovascular disease prevention were not at an increased risk of RCC (OR = 0.9, 95% CI = 0.6–1.4).
© 1999 Cancer Research Campaign

**Keywords:** renal cell cancer; analgesics; NSAID; aspirin; acetaminophen; phenacetin

Kidney cancer is a relatively rare malignancy. In the USA, there are roughly 30 000 new cases of kidney cancer each year, accounting for approximately 2% of all incident cancer cases diagnosed annually (Parker et al, 1996). Renal cell carcinoma (RCC) accounts for 80–85% of all kidney cancers in the USA. The remaining 15–20% of kidney cancers are mostly cancers of the renal pelvis, which are anatomically and histologically distinct from RCC (Devesa et al, 1990).

Chronic use of analgesics was first linked to the development of kidney cancer through a series of case reports which documented cancer of the renal pelvis occurring in heavy users of phenacetin (Bengtsson et al, 1968; Mahony et al, 1977). These uncontrolled observations were later confirmed by a number of case–control studies conducted in diverse populations (McCredie et al, 1982; McLaughlin et al, 1985; Jensen et al, 1989). In 1987, the International Agency for Research on Cancer concluded that there was sufficient evidence to name phenacetin as a human carcinogen.

The relationship between analgesic use and RCC is less clear. Until recently, only three case–control studies (Armstrong et al, 1976; McLaughlin et al, 1985; McCredie et al, 1988) and one cohort study (Paganini-Hill et al, 1989) had investigated the issue. Two of these studies (McLaughlin et al, 1985; McCredie et al, 1988) examined the relationship between phenacetin use and RCC risk, and consistently observed a positive exposure-disease association. Taken as a whole, the four studies were inconclusive with respect to non-phenacetin types of analgesics. Although human data are largely lacking, there is sufficient reason for concern in terms of the potential carcinogenic risk of non-phenacetin-based analgesics. Acetaminophen is a major metabolite of phenacetin in humans (Brodie and Axelrod, 1949), and is capable of inducing liver cell tumours in mice (Flaks and Flaks, 1983). Aspirin and most other non-steroidal anti-inflammatory agents (NSAIDs) are nephrotoxic in humans and in animals (Nanra, 1983). It is generally recognized that increased cell proliferation (which follows cytotoxicity and cell necrosis) is an important mechanism of increased cancer risk in humans (Henderson et al, 1991). Thus, nephrotoxic agents are potential nephrocarcinogens.

Given the public health importance of the possible analgesic–RCC association [in 1984, just prior to the start of this study, annual sales of analgesic drugs in the USA exceeded 1.9 billion dollars (Consumer Expenditure Study, 1985)], we initiated a large-scale case–control study of RCC in 1986 to address the relationship between sustained use of analgesics and RCC risk. This report describes in detail our findings with respect to individual classes of analgesics by formulation.

### MATERIALS AND METHODS

The study design and data collection have been described previously (Yuan et al, 1998*a*). In brief, the population-based cancer registry of Los Angeles County identified 1724 non-Asian patients, aged 25–74 years, with histologically confirmed RCC between April 1986 and December 1994. Among these, 301 patients died before we could contact them or were too ill to be

*Received 20 November 1998*
*Revised 21 March 1999*
*Accepted 23 March 1999*

*Correspondence to:* M Gago-Dominguez

**Table 1** Regular use of different subclasses of analgesics in relation to risk of renal cell carcinoma

| | Regular use[a] | Maximum weekly dose (g)[b] | | | |
|---|---|---|---|---|---|
| | | < 2 | 2 –< 4 | 4 –< 8 | 8+ |
| Aspirin | | | | | |
| Cases/controls | 413/323 | 98/118 | 102/81 | 99/63 | 105/56 |
| OR[c] | 1.6[g] | 1.1 | 1.6[g] | 2.0[g] | 2.4[g] |
| OR[d] (95% CI) | 1.5 (1.2–1.8) | 1.1 (0.8–1.6) | 1.5 (1.1–2.1) | 1.7 (1.2–2.5) | 1.9 (1.3–2.8) |
| Non-aspirin NSAID | | | | | |
| Cases/controls | 235/189 | 90/83 | 58/41 | 37/32 | 38/21 |
| OR[c] | 1.6[g] | 1.3 | 1.8[g] | 1.4 | 2.3[g] |
| OR[d] (95% CI) | 1.4 (1.1–1.8) | 1.3 (0.9–1.9) | 1.5 (1.0–2.3) | 1.3 (0.8–2.3) | 1.9 (1.1–3.5) |
| Acetaminophen | | | | | |
| Cases/controls | 300/209 | 67/66 | 63/47 | 83/57 | 79/36 |
| OR[c] | 1.9[g] | 1.3 | 1.8[g] | 1.9[g] | 2.8[g] |
| OR[d] (95% CI) | 1.7 (1.3–2.1) | 1.3 (0.9–1.9) | 1.7 (1.1–2.6) | 1.8 (1.2–2.6) | 2.1 (1.3–3.3) |
| Phenacetin | | | | | |
| Cases/controls | 86/55 | 41/37 | 22/6 | 23/12[e] | |
| OR[c] | 2.0[g] | 1.4 | 4.3[g] | 2.2[f] | – |
| OR[d] (95% CI) | 1.9 (1.3–2.7) | 1.3 (0.8–2.2) | 4.1 (1.5–10.8) | 2.3 (1.0–5.0) | |

[a]Defined as two or more times a week for 1 month or longer. [b]The sum may be slightly less than the total number of users due to the exclusion of subjects with missing values. [c]ORs as compared to non- or irregular users of analgesics (616 cases, 744 controls) after adjustment for level of education. [d]Further adjusted for usual body mass index (kg $m^{-2}$), history of hypertension (yes, no), average number of cigarettes smoked per day, current smoking status (smoker, non-smoker) and regular use of amphetamines (yes, no). [e]Included four cases and two controls whose maximum weekly dose of phenacetin was 8 or more g. [f]Two-sided $P < 0.05$ and [g] two-sided $P < 0.01$, test for OR = 1.0.

interviewed, and in 147 instances, the physician or patient refused to cooperate. Thus, we interviewed 1276 patients (74% of all eligible patients).

For each interviewed patient, we employed a standard algorithm to recruit a control who was matched to the patient on sex, date of birth (within 5 years), race and neighbourhood of residence at the time of cancer diagnosis. If a matched control could not be identified, the case was dropped from the study. Seventy-two cases were excluded from the study because of lack of matched controls. We completed in-person interviews on 1204 control subjects.

In-person, structured interviews were conducted in subjects' homes. The questionnaire requested information up to 2 years prior to the diagnosis of cancer for cases and 2 years prior to diagnosis of cancer of the index case for matched controls. The questionnaire requested information on demographic characteristics, height and weight, lifetime use of tobacco and alcohol, usual adult dietary habits, lifetime occupational history, prior medical conditions and prior use of medications.

We explicitly listed 44 over-the-counter and 33 prescription brand-name analgesics in the questionnaire (see Appendix). These brand-named drugs represent all common analgesics marketed in the USA since the 1950s. A picture album of the listed drugs was available to the respondents to assist in their recall of their usage. For each of the listed brand-name analgesics, we first asked the subjects whether they had ever taken the drug 20 or more times over their lifetime. If the answer was no, the subject was defined as a 'non-user'. Otherwise, the subjects was further asked if they had ever taken the drug two or more times a week for 1 month or longer. If the answer was no, subjects were classified as 'irregular user'. Otherwise, subjects were defined as 'regular user' and further asked about the ages at first and last use, duration of use, usual frequency and dosage of use, and the primary reason for such use. Aside from the 77 brand-name analgesics listed, the subjects were asked if they had taken any other analgesics regularly. If the answer was yes, the names of the analgesic drugs were recorded, along with ages at first and last use, duration of use, usual frequency and dosage of use, and the primary reason for use were similarly asked.

At the end of the interview, subjects were asked to give permission for us to contact all physicians who had prescribed analgesics to them. We attempted to contact all such physicians to validate the self-reported information on use of prescription analgesics, which included the name, dates and total months of use for every analgesic drug being prescribed.

The formulations of the listed analgesics as well as those volunteered by study subjects were established through numerous pharmaceutical sources, including the annually updated *Physician's Desk Reference*. Each active ingredient of brand-name analgesics was classified according to formulation as follows: aspirin, non-aspirin NSAID, acetaminophen, phenacetin and other. Similarly, each active ingredient of non-aspirin NSAID was further classified as propionic acid (such as ibuprofen and naproxen), acetic acid (such as indomethacin and sulindac), salicylic acid and 'other'. Age-specific exposure to a given drug was estimated from a subject's reported dose and duration of use at that age. Lifetime cumulative exposure (g) to a specific class of analgesics (such as aspirin) was computed by summing individual age-specific exposures across all ages and all brand-name drugs containing the active ingredient of interest. Lifetime cumulative exposure to all analgesics was computed by summing the cumulative exposures for aspirin, non-aspirin NSAID, acetaminophen, phenacetin and other analgesics. The average monthly dose (g) was calculated as lifetime cumulative dose divided by total months of use. Cumulative exposures and average monthly doses among regular users were grouped into quartiles according to their distributions among control subjects.

Data were analysed by standard matched-pair methods (Breslow and Day, 1980). Conditional logistic regression models were used to examine the relationship between analgesic use and RCC risk with adjustment for other risk factors identified in the

© 1999 Cancer Research Campaign 

**Table 2** Regular use of all analgesics in relation to risk of renal cell carcinoma

| All analgesics | Total | | | Males | | | Females | | |
|---|---|---|---|---|---|---|---|---|---|
| | Ca/Co | OR[a] | OR[b] (95% CI) | Ca/Co | OR[a] | OR[b] (95% CI) | Ca/Co | OR[a] | OR[b] (95% CI) |
| Regular use[c] | | | | | | | | | |
| No | 616/744 | 1.0 | 1.0 | 420/498 | 1.0 | | 196/246 | 1.0 | |
| Yes | 588/460 | 1.6[f] | 1.5 (1.3–1.8) | 361/283 | 1.5[f] | 1.4 (1.2, 1.8) | 227/177 | 1.8[f] | 1.7 (1.2–2.3) |
| Total consumption over lifetime (g)[d] | | | | | | | | | |
| < 60 | 115/112 | 1.3 | 1.3 (1.0–1.8) | 79/71 | 1.3 | 1.5 (1.0–2.1) | 36/41 | 1.2 | 1.2 (0.7–2.0) |
| 60 –< 312 | 125/102 | 1.5[f] | 1.5 (1.1–2.0) | 82/66 | 1.5[e] | 1.5 (1.0–2.2) | 43/36 | 1.6 | 1.6 (0.9–2.6) |
| 312 –< 1287 | 131/112 | 1.6[f] | 1.4 (1.0–1.9) | 87/75 | 1.5[e] | 1.2 (0.8–1.7) | 44/37 | 1.9[e] | 1.9 (1.1–3.2) |
| 1287+ | 205/121 | 2.2[f] | 1.9 (1.4–2.5) | 107/64 | 2.0[f] | 1.7 (1.2–2.5) | 98/57 | 2.5[f] | 2.1 (1.4–3.3) |
| Average monthly dose (g)[d] | | | | | | | | | |
| < 5 | 90/107 | 1.1 | 1.1 (0.8–1.6) | 61/68 | 1.1 | 1.2 (0.8–1.7) | 29/39 | 1.0 | 1.0 (0.5–1.8) |
| 5 –< 10 | 138/130 | 1.3 | 1.2 (0.9–1.6) | 99/88 | 1.3 | 1.2 (0.9–1.8) | 39/42 | 1.2 | 1.1 (0.6–1.9) |
| 10 –< 20 | 121/96 | 1.6[f] | 1.6 (1.1–2.2) | 76/60 | 1.6[e] | 1.5 (1.0–2.3) | 45/36 | 1.6 | 1.6 (0.9–2.8) |
| 20+ | 227/114 | 2.6[f] | 2.2 (1.6–2.9) | 119/60 | 2.3[f] | 2.0 (1.3–2.9) | 108/54 | 2.9[f] | 2.5 (1.6–3.9) |
| Maximum weekly dose (g)[d] | | | | | | | | | |
| < 2 | 115/136 | 1.1 | 1.1 (0.8–1.5) | 81/88 | 1.2 | 1.2 (0.8–1.7) | 34/48 | 1.0 | 1.0 (0.6–1.7) |
| 2 –< 4 | 136/120 | 1.3[e] | 1.3 (1.0–1.8) | 96/80 | 1.4 | 1.3 (0.9–1.8) | 40/40 | 1.2 | 1.3 (0.7–2.2) |
| 4 –< 8 | 134/99 | 1.7[f] | 1.6 (1.2–2.2) | 81/62 | 1.6[f] | 1.6 (1.1–2.4) | 53/37 | 1.9[f] | 1.6 (1.0–2.7) |
| 8+ | 195/93 | 2.7[f] | 2.3 (1.7–3.1) | 100/46 | 2.5[f] | 2.0 (1.3–3.1) | 95/47 | 3.0[f] | 2.6 (1.7–4.1) |
| Maximum No. of pills per day[d] | | | | | | | | | |
| < 1 | 47/57 | 1.1 | 1.1 (0.7–1.7) | 34/34 | 1.2 | 1.3 (0.7–2.2) | 13/23 | 0.9 | 0.9 (0.4–1.8) |
| 1 | 187/187 | 1.2 | 1.2 (0.9–1.6) | 130/129 | 1.2 | 1.2 (0.9–1.6) | 57/58 | 1.3 | 1.2 (0.7–2.0) |
| 2 | 118/91 | 1.7[f] | 1.6 (1.1–2.2) | 72/55 | 1.7[f] | 1.5 (1.0–2.4) | 46/36 | 1.7[e] | 1.6 (0.9–2.7) |
| 3+ | 228/113 | 2.6[f] | 2.2 (1.7–2.9) | 122/58 | 2.6[f] | 2.1 (1.4–3.1) | 106/55 | 2.7[f] | 2.4 (1.6–3.7) |

[a]Adjusted for level of education. [b]Further adjusted for usual body mass index (kg $m^{-2}$), history of hypertension (yes, no), average number of cigarettes smoked per day, current smoking status (smoker, non-smoker) and regular use of amphetamines (yes, no). [c]Defined as two or more times a week for 1 month or longer. [d]The sum may be slightly less than the total number of users due to the exclusion of subjects with missing values. [e]Two-sided $P < 0.05$ and [f]two-sided $P < 0.01$, test for OR = 1.0

present study (obesity, history of hypertension, cigarette smoking and regular use of amphetamines) (Yuan et al, 1998*a*, 1998*b*). Population attributable risks were computed as described in Breslow and Day (1980). All *P*-values quoted are two-sided.

### RESULTS

The mean age of the patients at diagnosis of RCC was 58.8 years. Most patients were non-Hispanic whites ($n$ = 1028) with the remaining being Hispanic whites ($n$ = 107) and African-Americans ($n$ = 69). On average, RCC patients had lower level of education than controls. The odds ratio (OR) for RCC was 0.6 (95% confidence interval (CI) = 0.5–0.7) for those who had attended college (13 years or more of schooling) compared with those who had a high school education or lower. Thus, all ORs presented below were adjusted for level of education (high school or less, college or above).

Relative to lifetime non-users of analgesics, irregular users of analgesics exhibited no increase in risk of RCC (OR = 1.0, 95% CI = 0.7–1.4). On the other hand, regular users of analgesics (at least twice a week for 1 month or longer) showed a statistically significant increase in risk relative to non- or irregular users (OR = 1.6, 95% CI = 1.4–1.9). Among regular users, mean duration of lifetime use was significantly longer in cases than in controls (96 months vs 81 months, $P$ = 0.035).

Table 1 presents the analgesic–RCC relationship separately for each of the four major formulation classes of analgesics – aspirin, non-aspirin NSAID, acetaminophen and phenacetin. There was a statistically significant elevation in risk of RCC associated with use of each of the four chemical classes of analgesics. Overall risk was very similar for phenacetin and acetaminophen formulations. Overall risk was also similar for aspirin and non-aspirin NSAID classes. The latter two risk estimates were modestly lower than for phenacetin and acetaminophen compounds, but the two sets of risks were not statistically different from each other. Within each class of analgesics, there was generally increasing risk with increasing level of exposure, irrespective of whether exposure was defined as maximum weekly dose (see Table 1), average monthly dose, maximum number of pills per day, or total intake over lifetime (data not shown). Adjustment for other risk factors for RCC did not materially alter any of the analgesic–cancer associations. Results in men were numerically and statistically comparable to those in women. We also conducted pair-wise comparisons of risks associated with per unit gram intake of aspirin, non-aspirin NSAID and acetaminophen with adjustment for the level of intake of other analgesics. No differences were observed.

We further examined the risk of RCC from non-aspirin NSAIDs according to their organic acid formulations. All formulations of non-aspirin NSAID examined were positively associated with RCC risk: the ORs after adjustment for other risk factors were 1.3 (95% CI = 1.0–1.7), 1.5 (95% CI = 1.0–2.2) and 1.7 (95% CI = 1.1–2.7) for propionic, salicylic and acetic acids respectively.

There were 159 cases and 133 controls who were exclusive users of aspirin, 61 cases and 50 controls who were exclusive users of non-aspirin NSAIDs, and 74 cases and 54 controls who were exclusive users of acetaminophen. There were no exclusive users of phenacetin due to the fact that all phenacetin-containing analgesics consumed by study subjects also contained aspirin.

*© 1999 Cancer Research Campaign*

**Table 3** Average daily dose and duration of use of aspirin and risk of renal cell carcinoma

| | ≤ 325[a] mg per day | | | | ≥ 326 mg per day | | | |
|---|---|---|---|---|---|---|---|---|
| | **Cases** | **Controls** | **OR[b]** | **OR[c] (95% CI)** | **Cases** | **Controls** | **OR[b]** | **OR[c] (95% CI)** |
| Aspirin only for cardiovascular disease prevention | 40 | 50 | 0.9 | 0.9 (0.6–1.4) | 14 | 9 | 2.0 | 2.0 (0.8–5.1) |
| Duration of use (years) | | | | | | | | |
| < 5 | 20[d] | 27 | 0.8 | 0.8 (0.4–1.4) | 4 | 4 | 1.4 | 1.7 (0.4–7.8) |
| ≥ 5 | 19 | 23 | 1.0 | 1.0 (0.5–1.9) | 10 | 5 | 2.6 | 2.3 (0.7–7.4) |
| Aspirin only for any reason | 77 | 91 | 1.0 | 1.0 (0.7–1.5) | 82 | 40 | 2.7[f] | 2.5 (1.6–3.9) |
| Duration of use (years) | | | | | | | | |
| < 5 | 41[d] | 50 | 1.0 | 1.0 (0.6–1.5) | 45 | 27 | 2.2[f] | 2.0 (1.2–3.4) |
| 5 –< 10 | 22 | 26 | 1.1 | 1.0 (0.5–1.9) | 14 | 7 | 2.9[e] | 3.0 (1.1–8.2) |
| ≥ 10 | 12 | 15 | 1.1 | 1.3 (0.5–3.0) | 23 | 6 | 4.9[f] | 4.3 (1.6–11.3) |

[a]One regular strength aspirin contains 325 mg of aspirin. [b]ORs as compared to non- or irregular users of analgesics (616 cases, 744 controls) after adjustment for level of education. [c]ORs were further adjusted for usual body mass index (kg $m^{-2}$), history of hypertension (yes, no), average number of cigarettes smoked per day, current smoking status (smoker, non-smoker) and regular use of amphetamines (yes, no). [d]The sum was slightly less than the total number of users due to the exclusion of subjects with missing values. [e]Two-sided $P < 0.05$ and [†] two-sided $P < 0.01$, test for OR = 1.0.

Compared with non- or irregular users of analgesics, increased risks of RCC were observed among exclusive users of aspirin (OR = 1.4; 95% CI = 1.1–1.9; *P* = 0.01), non-aspirin NSAID (OR = 1.5; 95% CI = 1.0–2.2; *P* = 0.06), and acetaminophen (OR = 1.6; 95% CI = 1.1–2.4; *P* = 0.03) after adjustment for other risk factors. Prescription analgesics frequently contain narcotic analgesics (such as codeine, propoxyphene, oxycodone and meperidine). There were only a few study subjects (six cases, seven controls) who exclusively used narcotic analgesics and no increased risk of RCC relative to non- or irregular users of analgesics was observed (OR = 1.0; 95% CI = 0.3–3.2).

There were 288 cases and 216 controls who used more than one chemical class of analgesics regularly. These regular users of ‘mixed analgesics’ exhibited comparable cancer risk level as users of single classes of analgesics (OR = 1.5, 95% CI = 1.2–1.9 when compared with non- or irregular users of analgesics).

Results in Table 1 indicated that RCC risk associated with per unit gram intake of aspirin consumed was comparable to that of acetaminophen, phenacetin and non-aspirin NSAIDs. Since it is biologically plausible that the carcinogenic potencies of analgesics are similar across the various formulations and this concept is supported by empirical data, we constructed overall analgesic exposure indices by summing the formulation-specific indices across categories of formulation. Such a cumulative exposure index also provides a reliable estimate of the contribution of analgesics categorically to RCC risk. Table 2 presents the results of these summary indices of analgesic use – total lifetime intake (g), average monthly dose (g), maximum weekly dose (g) and maximum number of pills per day. Independent of the exposure index under examination, there was statistically significant increase in cancer risk with increasing level of exposure. The risk associated with the highest level of exposure was at least 2.2-fold relative to no exposure. Similar results were obtained when adjustment was made for other risk factors for RCC. Results were similar between men and women (Table 2).

We examined the analgesic–RCC association according to the reason for drug use. Significantly elevated risks were observed among all regular users of analgesics except those who took analgesics for cardiovascular disease prevention (OR = 1.0; 95% CI = 0.7–1.6), or for cold, flu or allergy (OR = 1.1; 95% CI = 0.7–1.6). Subjects who took analgesics for cold, influenza or allergy usually used relatively low doses for short periods of time: the mean lifetime cumulative dose among cases was 0.8 kg, which was only 15% of that (5.5 kg) among cases who used the drug to alleviate headache.

Among regular users of analgesics who took the drug for the sole reason of cardiovascular health (all of them were exclusive users of aspirin), no increase in risk of RCC was observed among those taking 325 mg or less of aspirin per day (OR = 0.9; 95% CI = 0.6–1.4). On the other hand, there was a duration-dependent increase in risk among those taking higher doses of aspirin for this reason, although none of the ORs reached statistical significance (Table 3). This set of results was confirmed when we repeated the analysis on the larger set of subjects comprising all exclusive users of aspirin regardless of reason for use. Among those taking 325 mg or less of aspirin per day, no increase in risk of RCC was observed regardless of duration of use (up to 10 or more years). In contrast, those taking higher doses per day exhibited a statistically significant 2.5-fold risk relative to non- and irregular users of analgesics. Furthermore, this elevation in risk was duration-dependent (Table 3).

There were 578 individual prescription analgesics reported by cases and 384 by controls. Among cases, we received responses from physicians to 195 (34%) of these self-reported prescription analgesics, and 85 (44%) showed agreement on brand name between physician records and self-reported information. Among controls, the corresponding figures were 139 (36%) and 59 (42%) respectively.

### DISCUSSION

To our knowledge, the present study is the largest case–control study of RCC ever conducted in a single, geographically defined study population. The large sample size of this study allows for the effects of major classes of analgesics (acetaminophen, aspirin, non-aspirin NSAID and phenacetin) to be compared with a reasonable degree of certainty. Our data demonstrate that sustained use of analgesics is a significant, independent risk factor for RCC, and that the elevation in risk extends across major formulation categories including aspirin, other NSAIDs, acetaminophen and phenacetin.

© 1999 Cancer Research Campaign 

We were concerned that recall bias might explain the observed analgesic–RCC associations. We were able to indirectly address this issue through an analysis of an ongoing parallel study of bladder cancer in Los Angeles, which shared the same set of questions on medication use as the present study. Furthermore, the same team of interviewers was used in both studies. A total of 1379 bladder cancer patients and an equal number of age-, sex- and race-matched neighbourhood controls were included in this analysis. There were 498 (36%) bladder cancer patients and 537 (39%) control subjects who had used analgesics regularly. The risk of bladder cancer associated with regular use of analgesics was 0.9 (95% CI = 0.8–1.1).

Besides the current study, there is one other large-scale study that has addressed the analgesic–RCC association. This multicentre, international case–control study involved 1732 RCC patients and 2309 controls (McCredie et al, 1995). The investigators noted no increase in risk of RCC among regular users of phenacetin, acetaminophen and salicylates (mainly aspirin). Thus, results of this study are in rather stark contrast to those reported here. We have no clear explanation to these disparate findings. However, the design of our study and that of McCredie et al (1995) differs in several respects. Our target population was a single, geographically defined population consisting primarily of a single racial-ethnic group (non-Hispanic whites). All interviews were conducted by a single team of four interviewers and virtually all matched case–control pairs were interviewed by the same interviewer. Extensive research was conducted in the compilation of the brand-name analgesic list to ensure comprehensive coverage of the relevant time period. A picture album of the explicitly named analgesics was developed and made available to the respondents to aid in their recall. Finally, there was an attempt to validate all self-reported usage of prescription analgesics with physician records. Although only one-third of all self-reported prescription analgesic usage were successfully traced, we observed no difference in the rate of concordance between physician records and self-reports between the case and the control groups (44% and 42% respectively). In contrast, the multicentre study included subjects from five centres in four countries with varying patterns of analgesic use. Country-specific brand-names of analgesics were compiled, but different criteria were used to construct the drug lists across the various centres. No visual aid was offered to the respondents during the interviews to assist in recall and there was no attempt to validate self-reported usage of prescription medications.

Phenacetin has been found to induce renal cell adenomas and RCC in rodents (Johansson, 1981; Nakanishi et al, 1982). A number of case–control studies have shown regular use of phenacetin to be associated with a 1.5- to 6.0-fold increased risk of RCC (McLaughlin et al, 1985; Maclure and MacMahon, 1985; McCredie et al, 1988; Kreiger et al, 1993; Mellemgaard et al, 1994). The present study confirms that sustained use of phenacetin is a risk factor for RCC, conferring a two-fold excess risk among regular users of this drug. Phenacetin has been absent from all drugs manufactured in the USA since 1987.

Acetaminophen, a major metabolite of phenacetin, can induce renal proximal tubular necrosis (Emeigh Hart et al, 1991) and increase the incidence of kidney preneoplastic lesions and renal adenomas in carcinogen-treated rodents (Tsuda et al, 1984). Several case–control studies of RCC have investigated the specific relationship with acetaminophen exposure. Results are mixed. Two studies (McCredie et al, 1993; Derby and Jick, 1996) have reported dose-dependent, positive associations while others found no association (McLaughlin et al, 1985; Kreiger et al, 1993; McCredie et al, 1995; Rosenberg et al, 1998). The present study demonstrates that sustained use of acetaminophen is associated with a two-fold risk of RCC, with a statistically significant dose–response relation after adjustment for phenacetin use.

Aspirin in therapeutic doses can be nephrotoxic, producing renal tubular damage, papillary necrosis and chronic interstitial nephritis (Prescott, 1982). Experimental models have shown that chronic administration of aspirin can induce progressive toxic damage to the proximal convoluted tubules, renal papillary necrosis and chronic interstitial nephritis in rodents (Plummer et al, 1975; Prescott, 1982; Burrell et al, 1991). Human data regarding carcinogenesis are relatively sparse. One cohort study of a retirement community in Southern California found that daily aspirin use was associated with a sixfold risk of kidney cancer in men and a twofold risk in women (Paganini-Hill et al, 1989). A few case–control studies have reported use of aspirin to be related to an increased risk of RCC (Asal et al, 1988; Mellemgaard et al, 1994) while others found no such association (McLaughlin et al, 1985; McCredie et al, 1995). In the present study, exclusive users of aspirin who took dosages higher than 325 mg per day were clearly at an increased risk of RCC.

Experimental models have shown that chronic administration of various non-aspirin NSAIDs can induce papillary necrosis and chronic interstitial nephritis in rodents (Prescott, 1982). In addition, phenylbutazone, an NSAID, has been shown to induce adenomas and RCC in treated rats (Kari et al, 1995). In humans, acute tubular necrosis, papillary necrosis and interstitial nephritis have been histologically or radiographically confirmed in patients consuming therapeutic doses of non-aspirin NSAID (Adams et al, 1986; Segasothy et al, 1994). Due to the novelty of this class of compounds, no prior studies have investigated their carcinogenic potential in humans. The present study shows that sustained use of non-aspirin NSAID is related to RCC development in a dose-dependent manner.

Interestingly, our data implicate somewhat comparable levels of carcinogenic potency across the major classes of analgesics (aspirin, non-aspirin NSAID, phenacetin and acetaminophen) despite their chemical and pharmacological differences. The current findings suggest a common pathway in the oncogenic potential of these chemically diverse drugs. One such possible pathway is the shared ability of these drugs to induce renal damage leading to increased cell proliferation at the target site, a recognized mechanism of increased cancer risk in humans (Henderson et al, 1991). Aspirin, non-aspirin NSAID, phenacetin and acetaminophen used alone or in combination have been reported to be associated with chronic nephropathy, chronic renal failure or end-stage renal disease in several case–control studies (Pommer et al, 1989; Sandler et al, 1989; 1991; Perneger et al, 1994). In vivo and in vitro rodent models have shown that aspirin, non-aspirin NSAID and acetaminophen undergo in situ metabolic activation in the kidney with subsequent covalent binding of the highly reactive metabolites to tissue/microsomal proteins, leading to tubular necrosis (Mitchell et al, 1977; McMurtry et al, 1978; Kyle and Kocsis, 1985; Emeigh Hart et al, 1991). In an animal model of chronic analgesic nephropathy in which renal papillary necrosis was induced by the administration of bromoethylamine 2-hydrobromide in male rats, Gobé and Axelsen (1991) demonstrated increased cell proliferation in the tubular epithelium and in the interstitium of the renal cortex in kidneys that exhibited renal papillary necrosis.

© 1999 Cancer Research Campaign

Our data suggest that chronic intake of one regular-strength aspirin (325 mg) or less a day for cardiovascular health does not lead to an increased risk of RCC whereas higher daily doses do so. It is important to note that controlled trials have shown that 325 mg of aspirin every two days in disease-free subjects results in about a 40% decrease in risk of a first myocardial infarction (Steering Committee of the Physicians Health Study Research Group, 1989) and that doses higher than 325 mg per day in patients with a history of myocardial infarction do not result in greater protection from a second myocardial infarction compared to lower dosages (Antiplatelet Trialists' Collaboration, 1994).

A number of studies have shown that regular use of aspirin or non-aspirin NSAID is associated with a 40–50% reduction in colorectal cancer (Giovannucci et al, 1995). NSAIDs inhibit the synthesis of COX-2-derived prostaglandins which are implicated in colon carcinogenesis (Eberhart et al, 1994). It is important to note that NSAID-induced renal damage is believed to be due to the inhibition of COX-1-derived renal prostaglandins which are important for renal homeostasis (Sabatini, 1996). Ruffin et al (1997) noted that experimental human subjects ingesting no more than 324 mg of aspirin per day for 14 days exhibited statistically significant, 20% reduction in colorectal mucosal prostaglandin $E_2$ and $F_{2\alpha}$.

There is epidemiological evidence that regular intake of aspirin or other forms of NSAID is protective against Alzheimer's disease and multi-infarct dementia, which constitutes the second commonest type of dementia among elderly people in the USA (Meyer et al, 1989). A large number of case–control and cohort studies have shown that use of NSAID is associated with a 30–50% reduction in risk of Alzheimer's disease (McGeer et al, 1996). In a randomized trial involving multi-infarct dementia patients, those taking one regular-strength (325 mg) aspirin daily for 3 years showed statistically significant improvement in both cerebral perfusion values and cognitive performance scores relative to the control group at each of the three annual follow-up evaluations (Meyer et al, 1989).

To place the risks (RCC) and benefits (heart disease, colorectal cancer, Alzheimer's disease) of regular use of NSAID in an appropriate perspective, one needs to consider the comparative incidence of the aforementioned health outcomes in the USA. While RCC is a relatively rare malignancy (six per 100 000 non-Hispanic whites) (Yu et al, 1999), the corresponding rates for colorectal cancer, Alzheimer's disease and fatal ischaemic heart disease are 44, 95 and 204 per 100 000 respectively (Kokmen et al, 1993; Liu et al, 1998; National Center for Health Statistics, 1996). Our data provide no evidence that ingestion of a regular strength (325 mg) aspirin a day even for as long as 10 years leads to any increased risk of RCC. Given that there is no evidence of further protection from cardiovascular disease, colorectal cancer or Alzheimer's disease development with a daily aspirin dose higher than 325 mg per day, we would recommend a dosage of 325 mg aspirin per day or less for preventive health purposes.

In summary, this large-scale, population-based case–control study strongly implicates chronic use of analgesics as a causal factor in RCC development. We estimate that 18% (15% in men, 25% in women) of RCC in Los Angeles County, California can be attributed to this iatrogenic exposure.

### ACKNOWLEDGEMENTS

This study was supported by grants P01 CA17054 and R35 CA53890 from the US National Cancer Institute, Bethesda, MD. We thank Ms Susan Roberts, Mr Roger Mathison and Ms Kazuko Arakawa of the University of Southern California/Norris Comprehensive Cancer Center for their assistance in data collection and management.

### APPENDIX

### Over-the counter analgesics

APC, ASA compound, Bancaps, Momentum, Empirin compound without codeine, regular aspirin, extra strength aspirin, buffered aspirin, arthritis strength Bufferin, regular strength Tylenol, extra strength Tylenol, Cotylenol, generic acetaminophen, regular Anacin, arthritis pain formula Anacin, Anacin-3 regular strength or aspirin-free Anacin, Anacin-3 maximum strength or aspirin-free Anacin, Excedrin, Excedrin P.M. or aspirin-free Excedrin, extra strength Excedrin, regular strength Datril, extra strength Datril, Advil, Nuprin, Cama, Comtrex, Coricidin, Dristan or advanced formula Dristan, 4-way cold tablet, Nyquil, Robitussin night relief, Sine-aid, Sinutab, Triaminicin, Bromoseltzer, Midol, Pamprin, Vanquish, Alka seltzer, Tempra, Goody's headache powders, Stanbank, BC power or tablets and Doan's pills.

### Prescription analgesics

Clinoril, Motrin, Anaprox, Feldene, Empirin with codeine, aspirin with codeine, APC with codeine, Tylenol with codeine, Tylox, Darvocet, Darvon, Darvon compound, Indocin, Fiorinal, Percocet-5, Percodan, phenylbutazone (Azolid, Azolid-A, Butazolidin, Butazolidin-alka), Norgesic or Norgesic forte, Phenaphen, Arthralgen, Midrin, Wigraine, Buff-a-comp, Nalfon, Repan, Naprosyn, Valadol, Medache, Equagesic, Percogesic, Synalgos, Talwin or Talwin 50 and Talwin compound.

### REFERENCES

Adams DH, Howie AJ, Michael J, McConkey B, Bacon PA and Adu D (1986) Non-steroidal anti-inflammatory drugs and renal failure. *Lancet* **1**: 57–60

Antiplatelet Trialists' Collaboration (1994) Collaborative overview of randomised trials of antiplatelet therapy. I: Prevention of death, myocardial infarction, and stroke by prolonged antiplatelet therapy in various categories of patients. *Br Med J* **308**: 81–106

Armstrong B, Garrod A and Doll R (1976). A retrospective study of renal cancer with special reference to coffee and animal protein consumption. *Br J Cancer* **33**: 127–136

Asal NR, Geyer JR, Risser DR, Lee ET, Kadamani S and Cherng N (1988) Risk factors in renal cell carcinoma. II. Medical history, occupation, multivariate analysis, and conclusions. *Cancer Detect Prev* **13**: 263–279

Bengtsson U, Angervall L, Ekman H and Lehmann L (1968) Transitional cell tumors of the renal pelvis in analgesic abusers. *Scand J Urol Nephrol* **2**: 145–150

Breslow NE and Day NE (1980) *Statistical Methods in Cancer Research, Volume I: The Analysis of Case–Control Studies*. IARC Scientific publications 32, IARC: Lyon, France

Brodie BB and Axelrod J (1949) The fate of acetophenetidin (phenacetin) in man and methods for the estimation of acetophenetidin and its metabolites in biological material. *J Pharmacol Exp Ther* **97**: 58–67

Burrell JH, Yong JL and Macdonald GJ (1991) Analgesic nephropathy in Fischer 344 rats: comparative effects of chronic treatment with either aspirin or paracetamol. *Pathology* **23**: 107–114

Consumer Expenditure Study (1985) *Product Marketing for Beauty Industry Retailers and Manufacturers* **14**: 19–20

Derby LE and Jick H (1996) Acetaminophen and renal and bladder cancer. *Epidemiology* **7**: 358–362

© 1999 Cancer Research Campaign

Devesa SS, Silverman DT, McLaughlin JK, Brown CC, Connelly RR and Fraumeni JF Jr (1990) Comparison of the descriptive epidemiology of urinary tract cancers. *Cancer Causes Control* **1**: 133–141

Eberhart CE, Coffey RJ, Radhika A, Giardiello FM, Ferrenbach S and Dubois RN (1994) Up-regulation of cyclooxygenase 2 gene expression in human colorectal adenomas and adenocarcinomas. *Gastroenterology* **107**: 1183–1188

Emeigh Hart SG, Beierschmitt WP, Bartolone JB, Wyand DS, Khairallah EA and Cohen SD (1991) Evidence against deacetylation and for cytochrome P450-mediated activation in acetaminophen-induced nephrotoxicity in the CD-1 mouse. *Toxicol Appl Pharmacol* **107**: 1–15

Flaks A and Flaks B (1983) Induction of liver cell tumours in IF mice by paracetamol. *Carcinogenesis* **4**: 363–368

Giovannucci E, Egan KM, Hunter DJ, Stampfer MJ, Colditz GA, Willett WC and Speizer FE (1995) Aspirin and the risk of colorectal cancer in women. *N Engl J Med* **333**: 609–614

Gobé GC and Axelsen RA (1991) The role of apoptosis in the development of renal cortical tubular atrophy associated with healed experimental renal papillary necrosis. *Pathology* **23**: 213–223

Henderson BE, Ross RK and Pike MC (1991) Toward the primary prevention of cancer. *Science* **254**: 1131–1138

International Agency for Research on Cancer (1987) Analgesic mixtures containing phenacetin. *IARC Monographs on the Evaluation of Carcinogenic Risks to Humans. Updating of IARC Monographs* (Vol 1–42), pp. 310–312. IARC: Lyon, France

Jensen OM, Knudsen JB, Tomasson H and Sorensen BL (1989) The Copenhagen case-control study of renal pelvis and ureter cancer: role of analgesics. *Int J Cancer* **44**: 965–968

Johansson SL (1981) Carcinogenicity of analgesics: long-term treatment of Sprague-Dawley rats with phenacetin, phenazone, caffeine and paracetamol (acetamidophen). *Int J Cancer* **27**: 521–529

Kari F, Bucher J, Haseman J, Eustis S and Huff J (1995) Long-term exposure to the anti-inflammatory agent phenylbutazone induces kidney tumors in rats and liver tumors in mice. *Jpn J Cancer Res* **86**: 252–263

Kokmen E, Beard CM, O'Brien PC, Offord KP and Kurland LT (1993) Is the incidence of dementing illness changing? A 25-year time trend study in Rochester, Minnesota (1960–1984). *Neurology* **43**: 1887–1892

Kreiger N, Marrett LD, Dodds L, Hilditch S and Darlington GA (1993) Risk factors for renal cell carcinoma: results of a population-based case-control study. *Cancer Caus Contr* **4**: 101–110

Kyle ME and Kocsis JJ (1985) The effect of age on salicylate-induced nephrotoxicity in male rats. *Toxicol Appl Pharmacol* **81**: 337–347

Liu L, Deapen D, Bernstein L and Ross R (1998) *Cancer in Los Angeles County: Incidence and Mortality by Race/Ethnicity 1988–1995*. Los Angeles County Cancer Surveillance Program, University of Southern California: Los Angeles, CA

McCredie M, Ford JM, Taylor JS and Stewart JH (1982) Analgesics and cancer of the renal pelvis in New South Wales. *Cancer* **49**: 2617–2625

McCredie M, Ford JM and Stewart JH (1988) Risk factors for cancer of the renal parenchyma. *Int J Cancer* **42**: 13–16

McCredie M, Stewart JH and Day NE (1993) Different roles for phenacetin and paracetamol in cancer of the kidney and renal pelvis. *Int J Cancer* **53**: 245–249

McCredie M, Pommer W, McLaughlin JK, Stewart JH, Lindblad P, Mandel JS, Mellemgaard A, Schlehofer B and Niwa S (1995) International renal-cell cancer study. II. Analgesics. *Int J Cancer* **60**: 345–349

McGeer PL, Schulzer M and McGeer EG (1996) Arthritis and anti-inflammatory agents as possible protective factors for Alzheimer's disease: a review of 17 epidemiologic studies. *Neurology* **47**: 425–432

McLaughlin JK, Blot WJ, Mehl ES and Fraumeni JF Jr (1985) Relation of analgesic use to renal cancer: population-based findings. *Natl Cancer Inst Monogr* **69**: 217–222

Maclure M and MacMahon B (1985) Phenacetin and cancers of the urinary tract. *N Engl J Med* **313**: 1479–1480

McMurtry RJ, Snodgrass WR and Mitchell JR (1978) Renal necrosis, glutathione depletion, and covalent binding after acetaminophen. *Toxicol Appl Pharmacol* **46**: 87–100

Mahony JF, Storey BG, Ibanez RC and Stewart JH (1977) Analgesic abuse, renal parenchymal disease and carcinoma of the kidney or ureter. *Aust N Z J Med* **7**: 463–469

Mellemgaard A, Niwa S, Mehl ES, Engholm G, McLaughlin JK and Olsen JH (1994) Risk factors for renal cell carcinoma in Denmark: role of medication and medical history. *Int J Epidemiol* **23**: 923–930

Meyer JS, Rogers RL, McClintic K, Mortel KF and Lotfi J (1989) Randomized clinical trial of daily aspirin therapy in multi-infarct dementia. A pilot study. *J Am Geriatr Soc* **37**: 549–555

Mitchell JR, McMurtry RJ, Statham CN and Nelson SD (1977) Molecular basis for several drug-induced nephropathies. *Am J Med* **62**: 518–526

Nakanishi K, Kurata Y, Oshima M, Fukushima S and Ito N (1982) Carcinogenicity of phenacetin: long-term feeding study in B6c3fl mice. *Int J Cancer* **29**: 439–444

Nanra RS (1983) Renal effects of antipyretic analgesics. *Am J Med* **75**: 70–81

National Center for Health Statistics (1996) *Vital Statistics of the United States, 1992: Vol 2, Mortality, Part A*. Hyattsville MD: US Dept of Health and Human Services, Public Health Service, CDC: 1996. United States Department of Health and Human Services publication PHS 96-1101

Paganini-Hill A, Chao A, Ross RK and Henderson BE (1989) Aspirin use and chronic diseases: a cohort study of the elderly. *Br Med J* **299**: 1247–1250

Parker SL, Tong T, Bolden S and Wingo PA (1996) Cancer statistics, 1996. *CA Cancer J Clin* **46**: 5–27

Perneger TV, Whelton PK and Klag MJ (1994) Risk of kidney failure associated with the use of acetaminophen, aspirin, and nonsteroidal anti-inflammatory drugs. *N Engl J Med* **331**: 1675–1679

Plummer DT, Leathwood PD and Blake ME (1975) Urinary enzymes and kidney damage by aspirin and phenacetin. *Chem Biol Interact* **10**: 277–284

Pommer W, Bronder E, Greiser E, Helmert U, Jesdinsky HJ, Klimpel A, Borner K and Molzahn M (1989) Regular analgesic intake and the risk of end-stage renal failure. *Am J Nephrol* **9**: 403–412

Prescott LF (1982) Analgesic nephropathy: a reassessment of the role of phenacetin and other analgesics. *Drugs* **23**: 75–149

Rosenberg L, Rao RS, Palmer JR, Strom BL, Zauber A, Warshauer ME, Stolley PD and Shapiro S (1998) Transitional cell cancer of the urinary tract and renal cell cancer in relation to acetaminophen use (United States). *Cancer Caus Contr* **9**: 83–88

Ruffin MT, Krishnan K, Rock CL, Normolle D, Vaerten MA, Peters-Golden M, Crowell J, Kelloff G, Boland CR and Brenner DE (1997) Suppression of human colorectal mucosal prostaglandins: determining the lowest effective aspirin dose. *J Natl Cancer Inst* **89**: 1152–1160

Sabatini S (1996). Pathophysiologic mechanisms in analgesic-induced papillary necrosis. *Am J Kidney Dis* **28**: S34–S38

Sandler DP, Smith JC, Weinberg CR, Buckalew VM Jr, Dennis VW, Blythe WB and Burgess WP (1989) Analgesic use and chronic renal disease. *N Engl J Med* **320**: 1238–1243

Sandler DP, Burr FR and Weinberg CR (1991) Nonsteroidal anti-inflammatory drugs and the risk for chronic renal disease. *Ann Intern Med* **115**: 165–172

Segasothy M, Samad SA, Zulfigar A and Bennett WM (1994) Chronic renal disease and papillary necrosis associated with the long-term use of nonsteroidal anti-inflammatory drugs as the sole or predominant analgesic. *Am J Kidney Dis* **24**: 17–24

Steering Committee of the Physicians' Health Study Research Group (1989) Final report on the aspirin component of the ongoing Physicians' Health Study. *N Engl J Med* **321**: 129–135

Tsuda H, Sakata T, Masui T, Imaida K and Ito N (1984) Modifying effects of butylated hydroxyanisole, ethoxyquin and acetaminophen on induction of neoplastic lesions in rat liver and kidney initiated by N-ethyl-N-hydroxyethylnitrosamine. *Carcinogenesis* **5**: 525–531

Yu MC, Yuan J-M and Ross RK (1999) Epidemiology of renal cell carcinoma. *Carcinoma of the Kidney, Testis and Rare Urologic Malignancies: Innovations in Management*, pp. 3–13. Springer-Verlag: Berlin, Heidelberg

Yuan J-M, Castelao JE, Gago-Dominguez M, Ross RK and Yu MC (1998*a*) Hypertension, obesity and their medications in relation to renal cell carcinoma. *Br J Cancer* **77**: 1508–1513

Yuan J-M, Castelao JE, Gago-Dominguez M, Yu MC and Ross RK (1998*b*) Tobacco use in relation to renal cell carcinoma. *Cancer Epidemiol Biomarkers Prev* **7**: 429–433

*© 1999 Cancer Research Campaign*
